# Inflammation, Infectious Triggers, and Parkinson's Disease

**DOI:** 10.3389/fneur.2019.00122

**Published:** 2019-02-19

**Authors:** Elisa Caggiu, Giannina Arru, Sepideh Hosseini, Magdalena Niegowska, GianPietro Sechi, Ignazio Roberto Zarbo, Leonardo A. Sechi

**Affiliations:** ^1^Microbiology Section, Department of Biomedical Sciences, University of Sassari, Sassari, Italy; ^2^Department of Clinical, Surgical and Experimental Medicine, Neurological Clinic, University of Sassari, Sassari, Italy

**Keywords:** Parkinson's disease, neurodegenerative disease, neuroinflammation, immune system, alpha-synuclein, autoimmunity, microglia activation, autoantibodies

## Abstract

Parkinson's disease is a neurodegenerative disorder characterized by progressive loss of dopaminergic neurons of the substantia nigra pars compacta with a reduction of dopamine concentration in the striatum. The complex interaction between genetic and environmental factors seems to play a role in determining susceptibility to PD and may explain the heterogeneity observed in clinical presentations. The exact etiology is not yet clear, but different possible causes have been identified. Inflammation has been increasingly studied as part of the pathophysiology of neurodegenerative diseases, corroborating the hypothesis that the immune system may be the nexus between environmental and genetic factors, and the abnormal immune function can lead to disease. In this review we report the different aspects of inflammation and immune system in Parkinson's disease, with particular interest in the possible role played by immune dysfunctions in PD, with focus on autoimmunity and processes involving infectious agents as a trigger and alpha-synuclein protein (α-syn).

## Introduction

**Figure 1:**
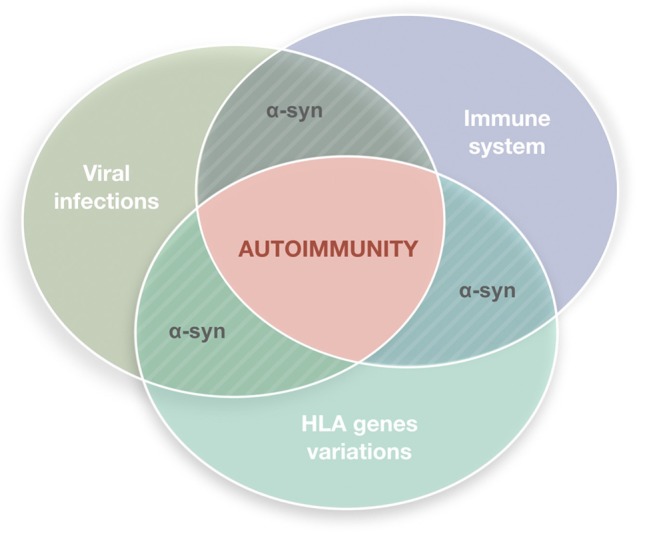
Autoimmune dysfunction in the etiology of Parkinson's disease (PD). The etiology of PD is multifactorial. It has been hypothesized that inflammation may underly the neurodegenerative process, with the immune system playing a key role. Viral infections are plausible triggers able to stimulate the immune system in genetically susceptible individuals inducing reactions that lead to autoimmune responses.

Parkinson's disease (PD) is a common disorder of the central nervous system (CNS) which determines postural instability, bradykinesia, resting tremor and muscle rigidity. The reduction of dopamine concentration in the striatum is related to the progressive death of neurons located on the substantia nigra pars compacta (SNpc) (1). Although many theories attempted to explain the causes of neuronal death in this region and to identify possible triggers, the exact PD etiology remains unknown. A growing body of evidence indicates that the nervous and immune systems act in synergy and maintain extensive communication (2–5). This interplay seems to underlie neuroinflammation which, apart from PD, is a constant feature of numerous neurodegenerative diseases such as Alzheimer's disease, dementia with Lewy bodies, amyotrophic lateral sclerosis, frontotemporal dementia or Huntington's disease (6) and may have multiple causes, including deficient regulation of immune responses associated with age advancement, infectious agents (bacteria or viruses), exotoxins (e.g., pesticides or MPTP), or deposition of insoluble protein fibrils (e.g., alpha-synuclein). In light of hypotheses seeing inflammation as the basis of neurodegenerative processes, dysfunction of the immune systems adds to the list of other PD contributors linking genetic mutations and environmental factors (Figure 1).

In this review, we aim at analyzing different aspects of inflammation and the immune system in PD providing a brief summary about the general characteristics of inflammatory responses with focus on a potential role of alpha-synuclein (α-syn), then moving forward to the analysis of innate immunity through an overview of microglial activity, and finally describing roles of the adaptive cell-mediated immunity in the disease. In addition, the hypothesis of PD as an autoimmune dysfunction is also discussed.

## Inflammation in PD

Already in 1988 McGeer's research team suggested that inflammation could be the first pathogenic mechanism of PD (7). At the same time, it has been observed that the use of non-steroidal anti-inflammatory drugs (NSAID) decreases the risk of PD, and this could be considered as a proof of inflammogenic characteristics of the disease (8). While neuronal death has been described as evidence of the ongoing CNS inflammation (9), several scientific reports documented microglial activation, cytokine production and the presence of autoantibodies univocally indicating inflammatory processes in PD (10–13). *In vitro* assays employing a dopaminergic neuron model showed some membrane proteins to be targeted by antibodies present in CFS of affected patients (14). A research performed on post-mortem excised brains revealed higher concentrations of cytokines and proapototic proteins in the striatum and cerebrospinal fluid (CSF) of PD patients compared to levels found in healthy controls, pointing at inflammation as a constant element of the disease (15). Through a further immunohistological study, McGeer et al. discovered several alterations in striatal microglial cells of patients with PD that appeared to be activated by an increased synthesis of proinflammatory cytokines (16). Nonetheless, it remains to be explained whether inflammation represents the first cause determining neurodegeneration or if it results from a selective damage process and cell degeneration.

Anthropogenic pollutants account for a significant part of neurotoxic agents. It's enough to think about 1-methyl-4-phenyl-1,2,3,6-tetrahydropyridine (MPTP) as the most striking example followed by certain pesticides released to the environment. MPTP, which may be accidentally produced during the manufacture of the analgesic opioid drug desmethylprodine (MPPP), causes irreversible neuronal damage and parkinsonian syndromes. Autopsies executed on subjects previously exposed to MPTP showed the activation of microglia persisting for even 16 years (17). These results provided a further evidence that an initial neuronal damage may lead to a neuroinflammatory process and have been confirmed by studies conducted on animal models, several of which demonstrated the ability of MPTP (18), rotenone insecticide (19, 20), and 6-hydroxydopamine (6-OHDA) (21) to activate microglial cells. In the same way, death of dopaminergic neurons has been observed both *in vitro* and *in vivo* after stimulation of microglia with lipopolysaccharides (LPS) (22–27).

## Alpha-Synuclein and Neuroinflammation in PD

A-syn is a soluble protein highly conserved among vertebrates, with α-helical lipid-binding motif common to all synucleins. Even though the physiological role of α-syn is not well understood, it is known to carry out crucial functions in synaptic plasticity (28) and in the release of neurotransmitters and synaptic vesicles (29, 30), thereby in regulating synaptic transmission through the stabilization of the SNARE protein complex, whose assembly and disassembly is essential for a correct membrane fusion on neuron terminals (30, 31). Consequently, α-syn is a key protein in the pathogenesis of PD. Although the scientific literature provides countless studies often yielding promising results, the reasons behind the accumulation of α-syn along with its causal role in neurodegeneration are still unresolved. However, it is ascertained that a higher expression of wild-type protein leads to formation of α-syn inclusions in neurons followed by cellular damage (32, 33).

According to post-mortem histological examinations of PD patients, alteration and aggregation of α-syn have been suggested to occur as an epiphenomenon probably mediated by other conditions, such as neuroinflammation (34). It has also been hypothesized that secreted extracellular α-syn can immediately activate glial cells and subsequently induce neuronal inflammation. Glial cells are able to capture and degrade α-syn masses in an effective way similar to neurons (35). The activation of microglia could encourage the production of some protective molecules including brain-derived neurotrophic factor (BDNF) but also proinflammatory cytokines, reactive oxygen and nitrogen species (36) which favor the progression of this neurodegenerative disease. In a study on murine models, Harms et al. observed the recruitment of peripheral innate immune cells such as monocytes and macrophage induced by injection of α-syn fibrils into the SNpc (37). Additionally, the authors found that the activation of MHC-II is as a primary step preceding the neurodegenerative process. Wild type α-syn is prone to post-translational nitrate modifications which enhance its propensity to aggregate (38). Moreover, nitrated α-syn, not recognized as a self-protein, can indirectly stimulate the maturation of harmful subsets of T helper lymphocytes capable of eliciting profound neural damages (39).

The maintenance of a perfect balance in the homeostasis of extracellular α-syn is essential for the wellbeing of the brain. Recently, a possible role of α-syn as a natural antimicrobial peptide (AMP) has been outlined. AMPs belong to an ancient family of proteins able to generate oligomers and fibrils similar to α-syn and constitute the first line of defense against pathogens acting as potent broad-spectrum antibiotics and immunomodulators (40). The expression of AMPs has not been confined to the brain but detected also in other tissues where the intervention of the adaptive immune system is limited (41). However, when dysregulated, the protective action of AMPs may lead to various toxic effects (42, 43). Some authors highlighted that α-syn exhibits antibacterial activity against *Escherichia coli* and *Staphylococcus aureus*, antifungal activity against pathogenic strains such as *Aspergillus flavus, Aspergillus fumigatus* and *Rhizoctonia solani*, and antiviral activity against West Nile Virus (WNV) (44, 45).

The alterations of bidirectional signaling within the gut-brain axis has been intensely studied in the context of the CNS inflammation involving microbial agents. Recently, *Proteus mirabilis* commonly overrepresented in the gut microbiota of PD mouse models has been shown to significantly induce motor deficits, to selectively cause dopaminergic neuronal damage and inflammation in substantia nigra and striatum, and to stimulate α-syn aggregation in the brains and colons of PD mice (46). The degree of acute and chronic inflammation in the intestinal wall has been positively correlated with the expression of α-syn in the enteric neurites of the upper gastrointestinal tract in pediatric patients (47).

The role of viral infections in diverging signaling pathways which regulate the establishment of innate immunity, such as those including proinflammatory molecules and DNA sensing, has been long hypothesized in PD pathogenesis. Herpes simplex virus 1 (HSV-1) encodes a ubiquitin-specific protease (UL36USP) which subverts type I IFN-mediated signaling, in particular IFN-β-induced signaling, independently from its deubiquitinase (DUB) activity (48). HSV-1 UL24 has the ability to inhibit the activation of IFN-β and interleukin-6 (IL-6) promoters mediated by cyclic GMP-AMP synthase (cGAS)—a newly identified foreign DNA sensor, and the interferon-stimulatory DNA-mediated IFN-β and IL-6 production during HSV-1 infection. Moreover, UL24 was shown to selectively block nuclear factor κB (NF-κB) without altering IFN-regulatory factor 3 promoter activation (49).

**Figure 2:**
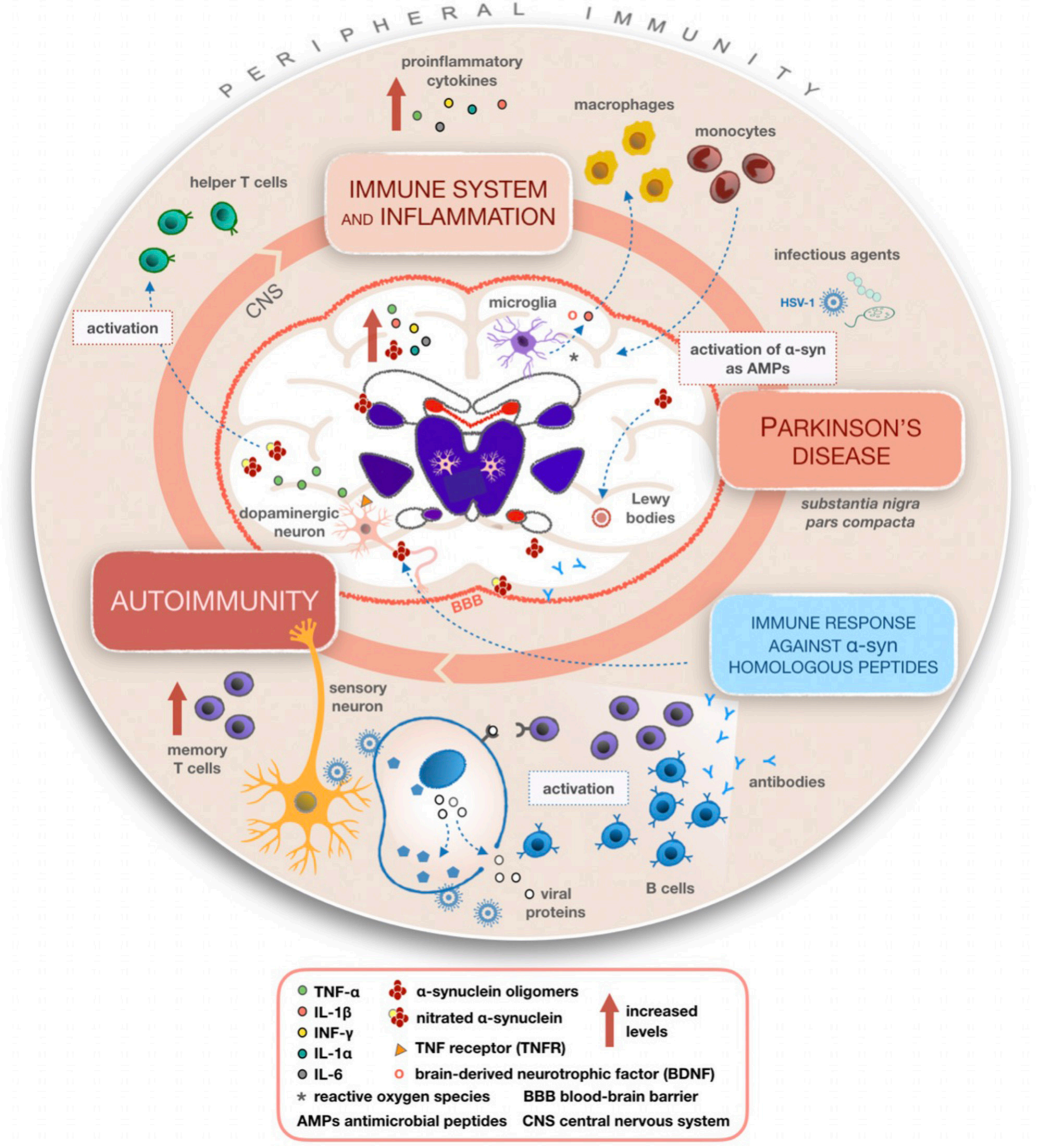
Mechanisms summarizing the involvement of inflammatory and immune processes in Parkinson's disease (PD). Once activated, microglial cells produce cytokines able to recruit macrophages and monocytes from peripheral compartments to the CNS, leading to altered peripheral immunity and various inflammatory processes within the CNS in PD patients. A possible mechanism of action giving rise to autoimmunity involves the reactivation of latent HSV-1 on infected sensory neurons and production of antibodies targeting alpha-synuclein (α-syn) fragments homologous to viral proteins. It is plausible that α-syn acting as an AMP becomes dysregulated during recurring infections with its consequent accumulation in the CNS.

Chronic neuroinflammation flanked by production of cytokines probably doesn't represent the initiating event of PD but, if lasting, this phenomenon could lead to disease progression through the involvement of microglia and astrocytes. It has been observed that cytokines such as TNF and IFN-γ have a high affinity to dopaminergic neurons (50, 51). In the CNS, these cytokines are mostly produced by microglia that could induce dopaminergic neurons with higher sensitivity (52). Several studies confirmed that PD patients display higher concentrations of TGF-β, IL-1β, IL-6, IFN-γ, and IL-1 in their CSF and striatum than the healthy controls (51, 53, 54). Similarly, a direct correlation between the raised levels of peripheral inflammatory cytokines and the degree of disability has been observed (55). According to a genetic screening for polymorphisms of DNA encoding proinflammatory cytokines such as IL-6, iNOS, IL-1β, and IL-1α (as shown in Figure 2), elevated quantities of these molecular mediators increase the risk of developing PD (56, 57). Schröder et al. (58) in their work reported increased levels of IL-2, IL-6, and TNFα and of the monocyte chemoattractant protein 1 (MCP-1) in the CSF of the PD patients whereas no differences were found in sera, confirming previous work (59).

## Innate Immunity in PD: Microglia Activation

Microglial cells are the principal actors of innate immunity in the CNS responsible for the protection and restoration of neurons (60). They can be activated by various external or internal insults such as neuronal dysfunction, trauma or certain toxin. Also, a wide range of molecules including viral or bacterial proteins, α-syn, cytokines and antibodies are able to induce the activation of microglia (61). Consequently, microglial cells produce different molecular mediators (e.g., reactive oxygen species, prostanoids and cytokines) with chemotactic and immunomodulatory functions. One of them is tumor necrosis factor (TNF) which in PD plays important roles contributing to the regulation of synaptic plasticity (62–64). PD brains are characterized by the presence of HLA-DR^+^ microglial cells and raised levels of CD68, an activation marker for microglia and macrophages, having a direct relation with α-syn aggregations and the duration of disease (7, 65). Moreover, an increased expression of MHC-II molecules in microglial cells has been observed in chronic neuroinflammation but not in the CNS of healthy subjects (66). Individuals with single nucleotide polymorphism (SNPs) at MCH-II locus are prone to develop PD, which indirectly proves the importance of adaptive immunity in these patients (67).

Microglia can be activated by numerous factors such as α-syn aggregates, neuromelanin, MMP-3, fibrinogen or environmental LPS toxins, MPTP, pesticides (rotenone, paraquat), proteasome and heavy metals, leading ultimately to neuroinflammation, and destruction of dopaminergic neurons (68). Studies employing positron emission tomography (PET) confirmed this phenomenon to occur in PD (7, 61, 69).

The activation of microglia and astrocytes by viruses has been shown to involve DNA-dependent activator of IFN regulatory factor (DAI) which specifically acts as an intracellular sensor for DNA viruses. DAI and its effector molecules are constitutively expressed in microgl cells and astrocytes with upregulation following viral challenge. In a DAI knockdown murine model, the release and production of neurotoxic mediators by HSV-1 challenged microglia and astrocytes was significantly attenuated. These findings suggest that DAI-mediated pathways may be crucial in the mechanisms of innate immunity activated against potentially lethal inflammation associated with neurotropic DNA virus infection (70).

## Adaptive Immunity: Activation of Cell-Mediated and Humoral Immunity in PD

The adaptive immune system shows specific responses against foreign antigens activating different T or B lymphocytes (71). The surveillance of homeostasis in the CNS is guaranteed by naïve and memory T cells (72, 73). T cell infiltration has been discovered in post-mortem brain sections of PD patients (74). The analysis of T cell subsets in peripheral blood mononuclear cells (PBMC) of affected patients showed altered immune responses and a decrease in the overall number of lymphocytes, but not in their frequency (75, 76). What is more, PD presents a particular immunological profile unseen in other neurological diseases (OND), where increased numbers of memory T cells and a reduced quantity of naïve T cells have been registered (77). As well, low CD4^+^:CD8^+^ ratio and a shift to more IFN-γ^−^ vs. IL-4-producing T cells have suggested the presence of cytotoxic T cell responses in PD patients (Figure 2) (75, 76, 78).

While a few specific proteins such as β-fibrinogen and transaldolase have been identified as possible biomarkers within T cells (79), it has been recorded that CD8^+^ subsets of PD subjects express Vβ8 receptors at lower frequency than healthy people (80). Moreover, several pathogenic alterations have been found in peripheral blood lymphocytes (PBL) of PD patients, for instance the presence of gaps in the DNA structure of lymphocytes and oxidation in purine b, high level of apoptosis, Cu/Zn superoxide dismutase activity, and the presence of micronuclei (81, 82). Interestingly, DNA damage has significantly declined after treatment with levodopa (83). Research on the overexpression of human α-syn through a recombinant adeno-associated virus vector serotype 2 (AVV2-SYN) system in SNpc of a murine model showed the infiltration of B and T cells alongside the activation of microglia suggesting that α-syn can recall the cells of adaptive immunity and stimulate inflammation (84). Recently, an important reduction in the number of T and B lymphocytes in mice knocked out for α-syn compared to wild type animals has been observed (85). A multiparameter flow cytometry analysis in patients with PD revealed a strong phenotypical shift of intrathecal monocytes and an elevated percentage of activated T lymphocytes coupled with an increase of proinflammatory cytokines in the CFS of PD patients (58).

Recently, Sulzer et al. (86) published a seminal work reporting selected peptides derived from two regions of α-syn which were highly recognized by specific T cell sets in PD patients. This response was predominantly mediated by IL-4 or IFNγ-producing CD4^+^ T cells, with likely contributions from CD8+/IFNγ producing T cells. Moreover, both α-syn epitopes originating from the natural processing of extracellular native α-syn present in blood and the fibrilized α-syn associated with PD triggered T cell responses. These epitopes were displayed by two MHC class II beta chain alleles, DRB5^*^01:01 and DRB1^*^15:01, associated with PD and by others not specific to PD (α-syn is not endogenously expressed by MHC class II expressing cells). The authors concluded that around 40% of the PD patients displayed immune responses to α-syn epitopes which may reflect varying trends in disease progression or impact from environmental factors.

Humoral immunity plays an important role in the etiopathogenesis of PD and many other neurodegenerative diseases. Given a reduction in the number of B cells as a frequent condition in PD patients (75, 87), it has been suggested that the proliferation of lymphocytes might be influenced by levodopa treatment, however some studies did not confirm such a correlation (76, 78). On the other hand, PD patients bear elevated levels of antibodies against dopamine (DA) neurons in comparison to healthy subjects (14, 88) while further investigations showed higher concentrations of antibodies targeting several peptides of α-syn and their homologs derived from HSV-1. It has been hypothesized that, in genetically predisposed individuals, previous HSV-1 infections may induce the production of autoantibodies through the molecular mimicry mechanism (13). Neurohistological studies disclosed the presence of immunoglobulins near dopaminergic neurons in the brains of patients with PD (89) which indicates a possible interaction between microglia and B lymphocytes. Finally, research on mouse models transfected with AVV-α-syn vector showed a significant deposition of IgG in the midbrain, suggesting humoral immunity to exert a remarkable function in the process of neurodegeneration in PD (84).

## Autoimmunity in PD

Environmental agents and the exposure to vectors (people, animals) may increase the risk of developing PD through transmission of viral infections or bacterial toxins. A case-control study conducted on a large number of PD patients proved a strong association between the disease and previous severe influenza, whereas an inverse association was observed regarding childhood infections, in particular red measles. Furthermore, an occupational exposure to domesticated animals increased the risk of PD (90). Viral infections most likely are not the primary cause but may act as triggers inducing the attack by the immune system against the CNS, dopaminergic neurons in particular. Numerous infectious agents are able to overcome the blood-brain barrier (BBB) and elicit inflammatory processes of the brain parenchyma, such as encephalitis. It is currently known that HSV-1 is one of the etiological agents responsible for sporadic viral encephalitis that often brings to neurological deficit in surviving patients. In murine models, HSV-1 determined a persistent viral lithic gene expression in ependyma during latency determining a chronic inflammatory response that the memory T cells were unable to counteract (91). Other studies in rodents showed that the H5N1 avian influenza virus passed the BBB inducing neurological signs, while a viral infection determined phosphorylation and aggregation of α-syn along with a substantial loss of dopaminergic neurons (92). An analogous study underlines that the highly pathogenic CA/09 H1N1 subtype was able to undermine microglial activation even without reaching the CNS (93). It is therefore conceivable that infectious agents do not act directly causing neuronal damage but, through secondary mechanisms such as the activation of the immune system trigger reactions leading to typical PD lesions. Other authors documented that people infected with hepatitis C virus (HCV) had a 30% greater likelihood of developing PD than healthy subjects (94). Similarly, a possible association between herpes simplex virus type 1 (HSV-1) infections and PD as higher antibody titers against HSV-1 were observed in the serum of PD patients but not in negative controls (95–97). This trend has been further confirmed through studies employing the micro-indirect hemagglutination (IHA) technique (98), however no increased production of antibodies against HSV-1 was observed in the CSF of PD when compared to controls (97, 98). The hypothesis that some viral triggers are related to the occurrence of CNS disorders such as Alzheimer's disease (AD) or PD has been further confirmed by investigations conducted *in vivo* (99, 100) and *in vitro* (101). The authors demonstrated that in cultured mouse cortical neurons, HSV-1 infection reduced the expression of synaptic proteins along with synaptic transmission through activation of glycogen synthase kinase (GSK)-3 and intracellular accumulation of amyloid beta protein (Aβ) determining synaptic dysfunctions which underlies cognitive impairment in AD. The above-mentioned findings have paved the way for a new branch of research aimed at unraveling the role of autoimmunity in PD and its implication in the loss of dopaminergic neurons typical to this pathology. Many efforts have been made in defining the extent to which autoimmunity is triggered by environmental variables, e.g., infective agents, metals, or other sources of inflammation. Cebrian and co-authors reported that human catecholaminergic substantia nigra and locus coeruleus neurons express MHC-I, therefore they may present antigens in response to exogenous agents and be particularly susceptible to T cell-mediated cytotoxic attack (102).

The importance of HSV-1 infection in triggering autoimmunity of PD has been further highlighted in connection with the mechanism of molecular mimicry and an immunologic cross-reactivity between HSV-1 and human α-syn leading in turn to the destruction of dopaminergic neurons of the substantia nigra (13). This study showed that the level of antibodies against HSV-1 peptides in PD patients was statistically higher than in healthy volunteers; the same trend was seen against human α-syn peptides homologous to viral epitopes. Similarly, molecular mimicry has been observed between a repeat region in the C-terminal half of the latent membrane protein 1 (LMP1) of Epstein-Barr virus (EBV) and the C-terminal region of α-syn. The authors hypothesized that antibodies directed against LMP1 present in genetically susceptible individuals cross-react with the homologous epitope on α-syn inducing its oligomerization (103).

A possible implication of HSV-1 in autoimmunity has been evaluated through another study conducted using the intracellular cytokine (ICC) method which showed that, alongside an alteration of cell patterns, the percentages of CD3, CD4, CD8, and CD56 lymphocytes were lower in PD patients compared to healthy subjects (87). The same authors reported the result of flow cytometry analysis which illustrates that human α-syn peptides and their HSV-1 homologs could remarkably induce the production of NK, CD4, CD8, and cells producing TNF-α in PD patients (87). The two homologous epitopes similarly stimulated T cell responses in a strongly correlated fashion. In addition, the immunogenic properties of these peptides were seen in cells secreting TNF-α which may play an important role in the pathogenesis of PD (87). In other studies, TNF-α exerted an effect on the plasticity of dopaminergic neurons which are particularly susceptible to this proinflammatory cytokine. The ligation of TNF-α with its receptors (TNFRs) is known to cause neuronal death under certain circumstances (62–64).

Further investigation confirmed the presence of autoimmune processes in PD without, however, indicating the triggering agents (86). Blood flow cytometry analysis performed in order to see how T cells respond against different α-syn portions showed a strong response against two specific peptides of this protein, namely Y39 and S129, in PD patients. In parallel, a relation between T cell responses and HLA risk alleles demonstrated that the main responses against α-syn epitope Y39 were expressed by four specific risk alleles. This study asserts the hypothesis that α-syn may activate T cell responses implicated in cell-mediated immunity, particularly autoimmunity, of PD.

A similar scenario is observed in the experimental autoimmune encephalitis model of multiple sclerosis (MS), as myelin proteins used to produce autoimmunity are not endogenous to MHC class II expressing cells but are accumulated and processed for MHC class II to be displayed by antigen presenting cells and microglia. In other autoimmune disorders, MHC class II response may precede MHC class I response (104). Moreover, as in T1D which features epitopes derived from both preproinsulin and additional proteins, it is plausible that PD-related epitopes derived from α-syn and supplementary peptides including molecules of infectious origin may be characterized by sequence homology (105). T cell responses in MS and T1D were shown to recognize self epitopes homologous to antigens from infectious microrganisms associated with the diseases. In MS, epitopes of EBV and *Mycobacterium avium* subsp. *paratuberculosis* homologous to IRF5 induced both humoral and cellular immune responses (106, 107).

It remains ambiguous whether autoimmunity is the primary cause or a consequence of the neurodegenerative process during progression of the disease. A substantial body of data suggest the possibility that autoimmunity may have an important role in the pathogenesis of PD and, if confirmed, a considerable revolution in terms of diagnostic and therapeutic approaches (e.g., immunotherapies and using T cells as biomarkers) should be expected in the near future.

## Author Contributions

EC, GA, and MN conceived the study and wrote the manuscript. SH performed bibliographic search. GS and IZ read the manuscript. LS conceived, organized the study, and critically reviewed the manuscript.

### Conflict of Interest Statement

The authors declare that the research was conducted in the absence of any commercial or financial relationships that could be construed as a potential conflict of interest.
